# Focal Herniation of Tibialis Anterior Muscle Diagnosed on Dynamic High-Resolution Ultrasonography Using Provocative Manoeuvres

**DOI:** 10.7759/cureus.17119

**Published:** 2021-08-12

**Authors:** Ravikanth Reddy

**Affiliations:** 1 Radiodiagnosis, St. John's Hospital, Bengaluru, IND

**Keywords:** focal muscle herniation, tibialis anterior, dynamic ultrasonography, myofascial defect, fencer’s lunge position

## Abstract

Focal protrusion of the muscle through an acquired or congenital myofascial defect is termed focal muscle herniation, and overall, tibialis anterior is the most commonly affected muscle. Tibialis anterior muscle typically herniates on attaining “fencer’s lunge” position. We report the case and describe the high-resolution ultrasonography appearances of focal tibialis anterior muscle herniation diagnosed using provocative manoeuvres in a 32-year-old female.

## Introduction

Focal muscle herniations are caused either due to a defect in the deep myofascial sheath secondary to trauma or due to the constitutional defects, commonly involving the lower extremities with tibialis anterior muscle being the most affected ones [1]. Diagnosis of focal muscle herniation can be challenging as the nodular swelling becomes apparent only on dynamic manoeuvres such as squatting or on the dorsiflexion of the leg that involves straining of the muscle and generally disappears at rest [2]. The above-described finding on dynamic ultrasonography is typical for focal muscle herniation. Prompt diagnosis of the entity on dynamic ultrasonography can help circumvent further imaging and unwanted invasive procedures, providing assurance to the patient simultaneously.

## Case presentation

**Figure 1:**
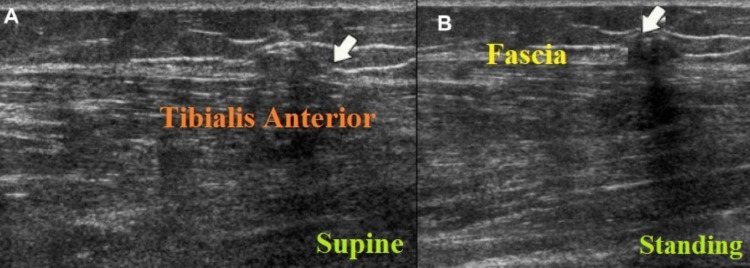
Longitudinal high-resolution ultrasonography images Longitudinal high-resolution ultrasonography image of the tibialis anterior muscle in supine position (arrow) demonstrating focal fascial defect (A). Longitudinal high-resolution ultrasonography image in weight-bearing standing position demonstrating focal herniation of hypoechoic muscle through the defect, with a mushroom-like appearance (arrow) consistent with features of focal muscle herniation (B).

A 32-year-old woman presented to the general surgery department with complaints of focal nodular soft-tissue swelling in the anterolateral aspect of the middle third of the left leg since 2 years. Moreover, the patient was able to increase or decrease the size of the nodular swelling based on her leg posture. She gave a history of blunt injury trauma to the lateral aspect of the left leg 4-5 years ago. On clinical examination, the nodular swelling was soft in consistency, compressible and nontender on palpation. The patient was referred for venous Doppler study of the left lower extremity as the surgeon has suspected a focal varicosity based on findings of the clinical examination. On venous Doppler, the saphenofemoral junction and saphenopopliteal junction were competent with normal valve closure times of common femoral, superficial femoral and popliteal veins. Moreover, there were no superficial varicosities or incompetent perforators in the left lower extremity. However, on high-resolution ultrasonography of the anterolateral aspect of the leg at the site of tenderness, there was a focal bulge in the contour of the tibialis anterior muscle, which became apparent on squatting or with the patient’s leg in dorsiflexion, and the focal bulge disappeared on supine position or with the patient’s leg in plantarflexion. The nodular lesion was located approximately 10 cm proximal to the lateral malleolus. A diagnosis of focal myofascial defect measuring 4.5-mm (mediolateral dimension) herniation of tibialis anterior muscle was made based on dynamic ultrasonography findings (Figures 1A, 1B). The patient was referred to the general surgery department where she was managed conservatively. The patient has given written informed consent to publish her case and clinical images.

## Discussion

Focal muscle herniations are classified based on aetiology as traumatic or constitutional. Traumatic muscle herniation may be due to direct or indirect injury. Direct injury is related to traumatic injury to the fascia, while indirect injury is related to the contracted muscle which causes disruption of the overlying fascia [3]. Constitutional muscle herniation may be due to a congenital defect in the fascia or due to a defect in the deep myofascial sheath secondary to physiological muscle hypertrophy causing elevated intracompartmental pressure, leading to fascial defects commonly located at the entry sites of the perforating vessels [4].

The most common sites of muscle herniation are the extremities; tibialis anterior muscle herniation in the lower leg constitutes the most frequent site because of the vulnerability of its fascia to trauma in the anterolateral tibial compartment [5]. Other muscles less frequently affected by muscle herniation in the lower extremity include gastrocnemius, peroneus longus, extensor digitorum longus and peroneus brevis [6]. On clinical examination, focal muscle herniations may present as a nodular soft-tissue swelling/focal mass in the subcutaneous plane.

Tibialis anterior muscle typically herniates when “fencer’s lunge” position is attained during the course of clinical examination or with dorsiflexion of the leg [7]. An increase in intracompartmental pressure within the anterior fascial compartment of the leg has been postulated to cause enhanced visualization of the tibialis anterior muscle herniation while squatting or standing, and vice versa, when the patient is lying supine. Although the clinical presentation provides a clue towards making a diagnosis of focal muscle herniation, dynamic ultrasonography has been regarded as a gold standard investigation for confirmation of diagnosis or to exclude alternative diagnoses such as muscle tears and tumours. Dynamic ultrasonography is of particular interest in the diagnosis of focal muscle herniations as they become more apparent during muscle contraction or after attaining a different posture.

Typical myofascial defect detected on dynamic ultrasonography is diagnostic for focal muscle herniation. Differential diagnoses for focal muscle herniation include varicosities, lipoma, focal rupture of muscle presenting as pseudohernia, angioma and arteriovenous malforma­tion [8]. However, these differentials mentioned demonstrate no size variability in changing the patient's position. Complications of muscle herniation may be related to pain, paresthesia and compartment syndrome [9]. Management of patients is usually conservative, and surgical procedures such as fasciotomy are reserved for cases with severe pain and nerve involvement [10].

## Conclusions

This case report describes the appearances of focal tibialis anterior muscle herniation caused by a defect in the deep fascial layer diagnosed on dynamic high-resolution ultrasonography using provocative manoeuvres and stresses on the fact that this rare clinical entity needs to be included in the differential diagnosis of nontender nodular soft-tissue swelling in the anterolateral aspect of the leg. Dynamic ultrasonography should be preferred as an imaging modality due to high diagnostic accuracy in diagnosing focal muscle herniations.
